# Description of *Klebsiella spallanzanii* sp. nov. and of *Klebsiella pasteurii* sp. nov.

**DOI:** 10.3389/fmicb.2019.02360

**Published:** 2019-10-25

**Authors:** Cristina Merla, Carla Rodrigues, Virginie Passet, Marta Corbella, Harry A. Thorpe, Teemu V. S. Kallonen, Zhiyong Zong, Piero Marone, Claudio Bandi, Davide Sassera, Jukka Corander, Edward J. Feil, Sylvain Brisse

**Affiliations:** ^1^Fondazione IRCCS Policlinico San Matteo, Unità Operativa Complessa Microbiologia e Virologia, Pavia, Italy; ^2^Scuola di Specializzazione in Microbiologia e Virologia, Università degli Studi di Pavia, Pavia, Italy; ^3^Biodiversity and Epidemiology of Bacterial Pathogens, Institut Pasteur, Paris, France; ^4^Department of Biology and Biochemistry, The Milner Centre for Evolution, University of Bath, Bath, United Kingdom; ^5^Infection Genomics, Wellcome Sanger Institute, Cambridge, United Kingdom; ^6^Department of Biostatistics, University of Oslo, Oslo, Norway; ^7^Center of Infectious Diseases, West China Hospital, Sichuan University, Chengdu, China; ^8^Department of Biosciences, University of Milan, Milan, Italy; ^9^Pediatric Clinical Research Center “Romeo ed Enrica Invernizzi”, University of Milan, Milan, Italy; ^10^Dipartimento di Biologia e Biotecnologie “L. Spallanzani”, Università di Pavia, Pavia, Italy; ^11^Department of Mathematics and Statistics, Helsinki Institute for Information Technology HIIT, University of Helsinki, Helsinki, Finland

**Keywords:** *Klebsiella oxytoca* complex, phylogeny, taxonomy, genome sequencing, *bla*_OXY_, MALDI-ToF mass spectrometry

## Abstract

*Klebsiella oxytoca* causes opportunistic human infections and post-antibiotic haemorrhagic diarrhea. This *Enterobacteriaceae* species is genetically heterogeneous and is currently subdivided into seven phylogroups (Ko1 to Ko4 and Ko6 to Ko8). Here we investigated the taxonomic status of phylogroups Ko3 and Ko4. Genomic sequence-based phylogenetic analyses demonstrate that Ko3 and Ko4 formed well-defined sequence clusters related to, but distinct from, *Klebsiella michiganensis* (Ko1), *K. oxytoca* (Ko2), *K. huaxiensis* (Ko8), and *K. grimontii* (Ko6). The average nucleotide identity (ANI) of Ko3 and Ko4 were 90.7% with *K. huaxiensis* and 95.5% with *K. grimontii*, respectively. In addition, three strains of *K. huaxiensis*, a species so far described based on a single strain from a urinary tract infection patient in China, were isolated from cattle and human feces. Biochemical and MALDI-ToF mass spectrometry analysis allowed differentiating Ko3, Ko4, and Ko8 from the other *K. oxytoca* species. Based on these results, we propose the names *Klebsiella spallanzanii* for the Ko3 phylogroup, with SPARK_775_C1^T^ (CIP 111695^T^ and DSM 109531^T^) as type strain, and *Klebsiella pasteurii* for Ko4, with SPARK_836_C1^T^ (CIP 111696^T^ and DSM 109530^T^) as type strain. Strains of *K. spallanzanii* were isolated from human urine, cow feces, and farm surfaces, while strains of *K. pasteurii* were found in fecal carriage from humans, cows, and turtles.

## Introduction

The genus *Klebsiella*, a member of the *Enterobacteriaceae* family, includes Gram-negative, non-motile (except *K. aerogenes*) and non-spore-forming capsulated bacteria. Bacteria belonging to the genus *Klebsiella* are found in water, soil and plants, and as commensals in the gut of animals including humans (Schmitz et al., 2002; Brisse et al., 2006; Caltagirone et al., 2017). In humans, *Klebsiella* species are frequently associated with hospital-acquired infections and are increasingly multidrug-resistant (Paczosa and Mecsas, 2018). *Klebsiella oxytoca* is the second most common *Klebsiella* species causing disease in humans, after *K. pneumoniae* (Broberg et al., 2014). *K. oxytoca* carries a chromosomally encoded β-lactamase gene (*bla*_OXY_) that confers resistance to amino- and carboxypenicillins (Fournier and Roy, 1997). This gene was shown to have diversified in parallel to housekeeping genes, and variants were classified into seven groups (*bla*_OXY–__1_ to *bla*_OXY–__7_) (Granier et al., 2003a, b; Fevre et al., 2005; Izdebski et al., 2015). *K. oxytoca* phylogenetic lineages were named Ko1, Ko2, Ko3, Ko4, Ko6, and Ko7 reflecting which *bla*_OXY_ variant they carry; note that Ko5 was not defined, as isolates carrying *bla*_OXY–__5_ represent a sublineage of Ko1 (Fevre et al., 2005). Taxonomic work has shown that *K. oxytoca* (*sensu lato*, i.e., as commonly identified in clinical microbiology laboratories) is in fact a complex of species, with *K. oxytoca* (*sensu stricto*) corresponding to phylogroup Ko2, *K. michiganensis* to Ko1 (Saha et al., 2013) and *K. grimontii* to Ko6 (Passet and Brisse, 2018). The closely related *K. huaxiensis* (Hu et al., 2019) represents yet another phylogroup, which we here denominate as Ko8 and which carries *bla*_OXY–__8_. Phylogroups Ko3, Ko4, Ko7, and *K. huaxiensis* were so far described only based on a single strain (Fevre et al., 2005; Hu et al., 2019), which has limited our ability to define their genotypic and phenotypic characteristics. While analyzing a large number of *Klebsiella* strains from multiple human, animal and environmental sources in and around the Northern Italian town of Pavia, we identified 3 Ko3, 13 Ko4, and 3 *K. huaxiensis* strains. The aim of this work was to define the taxonomic status of *K. oxytoca* phylogroups Ko3 and Ko4 and provide identification biomarkers for all members of the *K. oxytoca* species complex.

## Materials and Methods

### Bacterial Strains

Novel strains (3 Ko3, 13 Ko4, and 3 Ko8) were isolated through enrichment in Luria-Bertani broth supplemented with 10 μg/mL of amoxicillin, followed by isolation on Simmons citrate agar with 1% inositol (SCAI) medium (Van Kregten et al., 1984) and re-isolation on MacConkey agar. Additional strains, including type and reference strains of each *K. oxytoca* phylogroup and the type strain of *K. pneumoniae* (Brisse et al., 2014) were included in the study (Table 1). Strain SG271 (internal strain bank identifier, SB3356) and SG266 (SB3355) were included as reference strains for the phylogroups Ko3 and Ko4, respectively (Fevre et al., 2005).

**TABLE 1:** Strains included in the study, with provenance, and genomic information.

**Taxonomic designation**	**PhG^a^**	**Strain bank (SB) ID^b^**	**Strain name**	**Isolation year**	**Host**	**Source**	**Country**	**City**	**Accession no.**	**Intrinsic beta-lactamase^c^ (accession no.)**
*Klebsiella michiganensis*	Ko1	**SB4934**	W14 T (=CIP 110787 T)	2010	n.a.	Tooth brush holder	United States	Michigan	GCA_901556995	**OXY_1-7** (MN030558)
*K. michiganensis*	Ko1	**SB9**	16A079	1997	Human	Blood	Spain	Seville	GCA_901553745	OXY_1-2 (AY077484)
*K. michiganensis*	Ko1	**SB2908**	10A188	1997	Human	Blood	Italy	Genoa	GCA_901563895	OXY_5-1 (AJ871868)
*K. oxytoca*	Ko2	**SB175**	ATCC 13182 T	NA	NA	NA	NA	NA	GCA_900977765	OXY_2-2 (AF473577)
*K. spallanzanii*	Ko3	**SB6408**	SPARK_350_C1	2017	n.a.	Boot	Italy	Pavia	ERS3550822	**OXY_3-2** (MN030559)
*K. spallanzanii*	Ko3	**SB6411**	SPARK_775_C1 T (= CIP 111695T)	2017	Human	Urine	Italy	Pavia	ERS3550824	**OXY_3-3** (MN030560)
*K. spallanzanii*	Ko3	**SB6419**	SPARK_1442_C2	2018	Cow	Feces	Italy	Valle Salimbene	ERS2601707	**OXY_9-1** (MN030564)
*K. spallanzanii*	Ko3	**SB3356**	SG271	2000	Human	Peritoneal fluid	France	Paris	GCA_901563875	OXY_3-1 (AF491278)
*K. pasteurii*	Ko4	**SB3355**	SG266	2000	Human	Wound	France	Paris	GCA_901563825	OXY_4-1 (AY077481)
*K. pasteurii*	Ko4	**SB6407**	SPARK_327_C1	2017	Cow	Feces	Italy	Pavia	ERS3550826	OXY_4-1 (AY077481)
*K. pasteurii*	Ko4	**SB6410**	SPARK_613_C1	2017	Turtle	Feces	Italy	Sant’Alessio con Vialone	ERS2600949	OXY_4-1 (AY077481)
*K. pasteurii*	Ko4	**SB6412**	SPARK_836_C1 T (= CIP 111696T)	2017	Human	Feces	Italy	Pavia	ERS3550825	**OXY_4-2** (MN030561)
*K. pasteurii*	Ko4	**SB6424**	SPARK_1489_C1	2018	n.a.	Soil	Italy	San Genesio	ERS2601773	OXY_4-1 (AY077481)
*K. pasteurii*	Ko4	**SB6409**	SPARK_534_C3	2017	Turtle	Feces	Italy	Sant’Alessio con Vialone	ERS3550823	OXY_4-1 (AY077481)
*K. pasteurii*	Ko4	**SB6413**	SPARK_1058_C2	2018	Human	Feces	Italy	Pavia	ERS2601251	OXY_4-1 (AY077481)
*K. pasteurii*	Ko4	**SB6414**	SPARK_1260_C1	2018	Cow	Feces	Italy	Magherno	ERS2601488	**OXY_4-3** (MN030562)
*K. pasteurii*	Ko4	**SB6415**	SPARK_1268_C1	2018	Cow	Milk	Italy	Magherno	ERS2601499	**OXY_4-3** (MN030562)
*K. pasteurii*	Ko4	**SB6416**	SPARK_1269_C1	2018	Cow	Milk	Italy	Magherno	ERS2601500	OXY_4-1 (AY077481)
*K. pasteurii*	Ko4	**SB6417**	SPARK_1286_C1	2018	Human	Feces	Italy	Pavia	ERS2601525	**OXY_4-4** (MN030563)
*K. pasteurii*	Ko4	**SB6420**	SPARK_1445_C1	2018	Cow	Feces	Italy	Valle Salimbene	ERS2601710	OXY_4-1 (AY077481)
*K. pasteurii*	Ko4	**SB6423**	SPARK_1448_C2	2018	Cow	Feces	Italy	Valle Salimbene	ERS2601714	**OXY_4-5** (MN030567)
*K. pasteurii*	Ko4	**–**	SPARK_1531_C2	2018	n.a.	Water	Italy	Lardirago	ERS2601825	OXY_4-1 (AY077481)
*K. grimontii*	Ko6	**SB73**	06D021 T	1997	Human	Wound	France	Lille	GCA_900200035	OXY_6-1 (AJ871873)
*K. huaxiensis*	Ko8	**SB6421**	SPARK_1445_C2	2018	Cow	Feces	Italy	Valle Salimbene	ERS2601711	**OXY_8-2** (MN030565)
*K. huaxiensis*	Ko8	**SB6422**	SPARK_1448_C1	2018	Cow	Feces	Italy	Valle Salimbene	ERS2601714	**OXY_8-3** (MN030566)
*K. huaxiensis*	Ko8	**SB6425**	SPARK_1495_C1	2018	Human	Feces	Italy	Pavia	ERS2601786	OXY_8-1 (WP_112215366)
*K. huaxiensis*	Ko8	**SB6550**	WCHKl090001 T	2017	Human	Urine	China	Chengdu	GCA_003261575	OXY_8-1 (WP_112215366)

*NA, information not available; n.a. not applicable; T, type strain. ^*a*^PhG, *K. oxytoca* phylogroup. ^*b*^Internal strain collection number of the Biodiversity and Epidemiology of Bacterial Pathogens unit, Institut Pasteur. ^*c*^Bold characters represent the new OXY beta-lactamases submitted to the nomenclature database at https://bigsdb.pasteur.fr/klebsiella/klebsiella.html.*

### Genome Sequencing and Analyses

Colonies from the novel strains grown on MacConkey agar were collected and resuspended in distilled water for DNA purification, which was performed using QIAsymphony automated instrument with the kit QIAsymphony DSP Virus/Pathogen following the manufacturer’s recommendation. DNA was stored at −20°C until sequencing on an Illumina HiSeq X Ten platform with a 2 × 150 nt paired-end protocol. Reads were assembled using SPAdes v3.11 and the assemblies were annotated using Prokka v1.12 (Seemann, 2014). JSpeciesWS (Richter et al., 2016) was used to calculate the average nucleotide identity (ANI) using the BLAST algorithm (ANIb), whereas *in silico* DNA-DNA hybridization (isDDH) was performed through GGDC tool^1^ (formula 2) (Meier-Kolthoff et al., 2013). Sequences of *gyrA* and *rpoB* genes were obtained from genome assemblies using BLASTN, while 16S rRNA gene sequences were obtained using Barrnap^2^. The chromosomal *bla*_OXY_ sequences were also extracted, and the new amino-acid sequence variants were submitted to the Institut Pasteur MLST nomenclature database^3^ for variant number attribution, and to NCBI for accession number attribution. 16S rRNA, g*yrA*, *rpoB*, and *bla*OXY beta-lactamase gene sequences were aligned using Muscle (Edgar, 2004), concatenated (in the case of *rpoB* and *gyrB*) and phylogenetic relationships were assessed using MEGA v7.0 (Kumar et al., 2016). Genetic distances were inferred using the neighbor-joining method with the Jukes-Cantor correction (Jukes and Cantor, 1969) in the case of nucleotide sequences or maximum-likelihood with Jones-Taylor-Thornton (JTT) (Jones et al., 1992) model in the case of the beta-lactamase protein sequences. The genome-based phylogenetic analysis was performed on the concatenation of 3,814 core genes defined using Roary v3.12 (Page et al., 2015) with a BLASTP identity cut-off of 80% and presence in more than 90% of the isolates. *K. pneumoniae* ATCC 13883^T^ (GCA_000742135.1) was used as outgroup. An approximate maximum-likelihood phylogenetic tree was inferred using FastTree v2.1 (Price et al., 2010).

### Biochemical and Proteomic Analyses

A representative subset of strains (*n* = 30, 7 Ko1, 5 Ko2, 4 Ko3, 5 Ko4, 6 Ko6, and 3 Ko8) of phylogroups of the *K. oxytoca* complex was subjected to API20E (BioMérieux) and to phenotype microarray characterization using plates PM1 and PM2 (Biolog, Hayward, CA, United States) in aerobic conditions as previously described by Blin et al. (2017). The same subset of strains was also used to perform a MALDI-ToF mass spectrometry (MS) analysis following the protocol described by Rodrigues et al. (2018). Briefly, cell extracts were spotted onto an MBT Biotarget 96 target plate, air dried and overlaid with 1 μL of a saturated α-cyano-4-hydroxycinnamic acid (HCCA). Mass spectra were acquired on a Microflex LT mass spectrometer (Bruker Daltonics, Bremen, Germany) using the default parameters, preprocessed (applying smoothing and baseline subtraction) with FlexAnalysis software, and then imported and analyzed in a dedicated BioNumerics v7.6 (Applied-Maths, Belgium) database.

## Results

**FIGURE 1:**
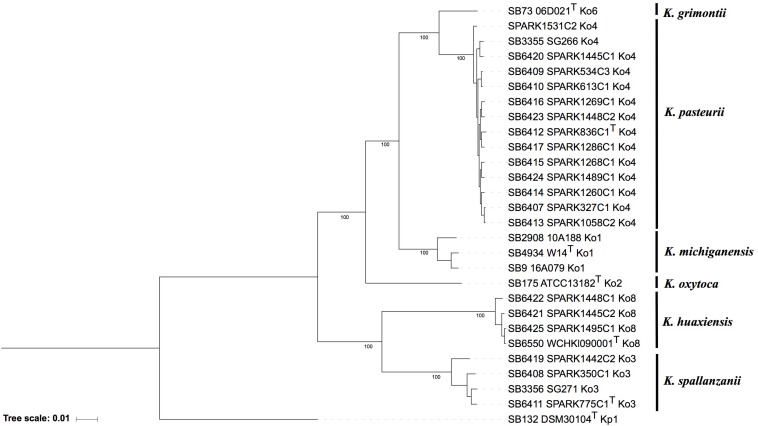
Maximum likelihood phylogenetic tree inferred based on the concatenated nucleotide sequence alignments of 3,814 core genes. The tree was rooted using *K. pneumoniae* DSM 30104^T^ (=ATCC 13883^T^). Taxonomic groups are indicated in front of the branches. Branch lengths represent the number of nucleotide substitutions per site (scale, 0.01 substitution per site). Bootstrap values are indicated at major nodes. Strain labels are given as Strain Bank ID (e.g., SB73) followed by original strain name, followed by the phylogroup. A “T” after the strain name indicates that the strain is the type strain of its taxon.

The phylogenomic analysis based on the concatenation of 3,814 core genes (Figure 1) showed six distinct and highly supported branches. The thirteen Ko4 strains were clustered with Ko4 reference strain SG266 (SB3355) and this group was related to, but clearly distinct from, *K. grimontii* (Ko6). The three Ko3 strains (SPARK_350_C1, SPARK_775_C1 and SPARK_1442_C2) formed a well-defined cluster with Ko3 reference strain SG271 (SB3356, Figure 1), whereas the remaining three strains (SPARK_1445_C2, SPARK_1448_C1, SPARK_1495_C1) clustered with *K. huaxiensis*, which formed a distinct phylogroup that we here name Ko8. We therefore identified novel strains of these three phylogroups, which were each previously recognized based on a single strain. Furthermore, genome-based phylogeny revealed that Ko4 shares a common ancestor with *K. grimontii*, *K. michiganensis* and *K. oxytoca*, whereas Ko3 and *K. huaxiensis* share a common ancestor distinct from the Ko1/Ko4/Ko6 one (Figure 1).

**FIGURE 2:**
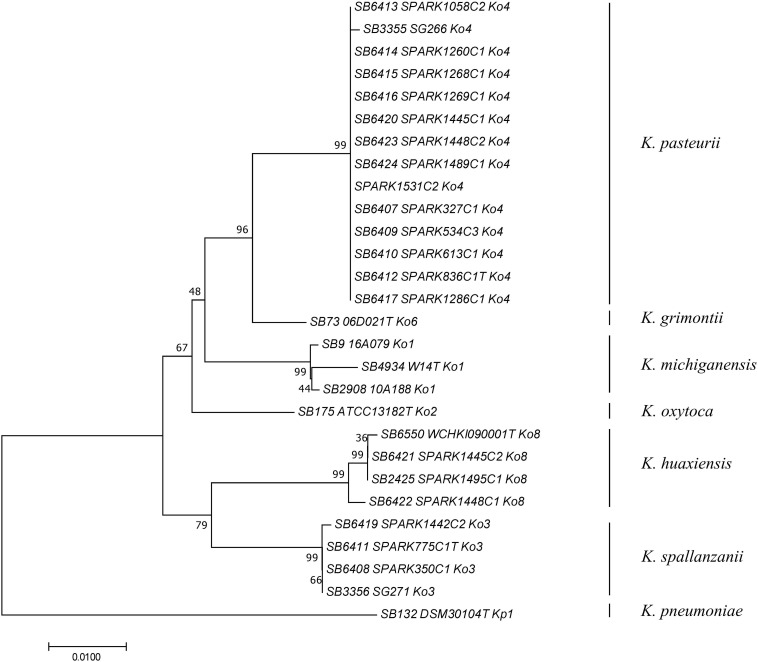
Phylogenetic relationships (neighbor-joining method, Jukes-Cantor correction) based on the concatenated sequences of *gyrA* and *rpoB* genes. The tree was rooted using *K. pneumoniae* DSM 30104^T^ (=ATCC 13883^T^). Taxonomic groups are indicated in front of the branches. Bootstrap proportions obtained after 1000 replicates are indicated at the nodes. Branch lengths represent the number of nucleotide substitutions per site (scale, 0.01 substitution per site). Strain labels are given as Strain Bank ID (e.g., SB73) followed by original strain name, followed by phylogroup. A “T” after the strain name indicates that the strain is the type strain of its taxon.

To determine how previously used phylogenetic markers (Brisse and Verhoef, 2001; Granier et al., 2003a, b; Fevre et al., 2005) would group these novel strains, the sequences of internal portions of the housekeeping genes *gyrA* (383 nt) and *rpoB* (501 nt), as well as the *rrs* (1,454 nt) sequence coding for 16S rRNA, were extracted from genomic sequences and compared to previously characterized sequences of reference and type strains from the *K. oxytoca* complex (Table 1). The clustering of Ko4 strains and Ko3 strains was supported by phylogenetic analysis of combined *gyrA* and *rpoB* gene sequences (Figure 2), as well as by single gene phylogenies (Supplementary Figures S1, S2), showing that either gene used alone would allow reliable identification. The phylogeny of the chromosomal OXY beta-lactamase gene (Supplementary Figure S3) was also in concordance with previous phylogenetic analyses. However, phylogroup Ko1 and Ko3 each harbored two different types of *bla*_OXY_, coding for OXY-1/OXY-5 and OXY-3/OXY-9, respectively (Supplementary Figure S3). As previously reported (Boye and Hansen, 2003; Naum et al., 2008; Passet and Brisse, 2018), the phylogeny based on the *rrs* gene was not reliable for species or phylogroup identification (type strain sequences were >97.8% similar), with only a few informative variable sites (Supplementary Figure S4).

Average nucleotide identity was estimated between Ko3 and Ko4, and the type strains of species of the *K. oxytoca* complex (Table 2). The three Ko3 strains, including SPARK_775_C1^T^, shared high identity (above 98%) with the Ko3 strain SG271 (SB3356) (data not shown). The ANI values of SPARK_775_C1^T^ (Ko3) strain with *K. huaxiensis* (WCHKl090001^T^), *K. michiganensis* (W14^T^), *K grimontii* (06D021^T^) and *K. oxytoca* (ATCC 13182^T^) were 90.7, 88.4, 88.3, and 87.9%, respectively (Table 2). The novel Ko4 strains showed approximately 98% ANI with Ko4 strain SG266 (SB3355). The ANI values of SPARK_836_C1^T^ (Ko4) with *K. grimontii*, *K. michiganensis, K. oxytoca* and *K. huaxiensis* were 95.5, 93.3, 90.6, and 87.1%, respectively (Table 2). Finally, the three Ko8 strains presented ANI values >99% with the type strain of *K. huaxiensis* (WCHKl090001^T^), showing that they belong to this recently described species. The isDDH relatedness range between the Ko3 and Ko4 type strains and other species was 36.3–44.1% and 34.3–67.8%, respectively. In conclusion, both ANI and isDDH values were below the thresholds proposed (Rossello-Mora and Amann, 2015) for species distinction (∼95–96% in the case of ANI, ∼70% in the case of isDDH), indicating that Ko3 and Ko4 represent two new species.

**TABLE 2:** Average nucleotide identity (ANI) values obtained among the type strains of members of the *Klebsiella oxytoca* complex.

			**Average nucleotide identity of test genome against query genomes**					
**Query genome^a^**	**Size (nucleotides)**	**DNA G + C content (mol %)**	**Ko1**	**Ko2**	**Ko3**	**Ko4**	**Ko6**	**Ko8**
Ko1	6 193 009	56.0	^∗^	91.65	88.53	93.09	93.23	87.47
Ko2	5 672 774	55.1	91.92	^∗^	88.22	90.81	91.06	87.05
Ko3	6 186 380	53.3	88.4	87.9	^∗^	87.99	88.32	90.7
Ko4	6 006 767	55.3	93.29	90.61	88.12	^∗^	95.52	87.11
Ko6	6 168 876	55.4	93.27	90.9	88.5	95.56	^∗^	87.45
Ko8	6 206 993	53.3	87.07	86.64	90.58	86.78	87.07	^∗^

*^*a*^Ko1, *K. michiganensis* W14^*T*^; Ko2, *K*. *oxytoca* ATCC13182^*T*^; Ko3, *K. spallanzanii* SPARK_775_C1^*T*^; Ko4, *K. pasteurii* SPARK_836_C1^*T*^; Ko6, *K. grimontii* 06D021^*T*^; Ko8, *K. huaxiensis* WCHKl090001^*T*^. ^∗^ is used when a genome is compared with itself.*

The phenotypic characteristics of Ko3 and Ko4 strains were analyzed and compared with those of other *Klebsiella* isolates. We confirmed that all strains were non-motile by microscopy and that all isolates were positive for indole, lactose, mannitol, malonate, lysine decarboxylase, and the ONPG test, and reduced nitrate to nitrite, whereas they were all negative for ornithine decarboxylase. Ko8 and Ko3 isolates were negative for Voges–Proskauer test and Ko3 isolates were urease positive (similar to Ko2). To define further the biochemical features of the *K. oxytoca* phylogroups, their carbon source utilization profiles were analyzed. Among 190 substrates, several appeared useful for differentiating the phylogroups among themselves and to differentiate Ko3 and Ko4 strains from other groups (Table 3 and Supplementary Figure S5). The inability to metabolize L-proline and tricarballylic acid differentiated Ko3 strains from other phylogroups except Ko8, which can be differentiated based on its unique ability to utilize 3-O-methyl-glucose. Ko4 had a weak but unique capacity to utilize glyoxylic acid, and differed from Ko6 (*K. grimontii*) by its inability to metabolize D-melezitose; Ko4 was otherwise similar to Ko6 for many features, consistent with their phylogenetic association.

**TABLE 3:** Differential biochemical characteristics of the taxa under study.

	***K. michiganensis***	***K. oxytoca***	***K. spallanzanii***	***K. pasteurii***	***K. grimontii***	***K. huaxiensis***
	**(Ko1, *n* = 7)**	**(Ko2, *n* = 5)**	**(Ko3, *n* = 4)**	**(Ko4, *n* = 5)**	**(Ko6, *n* = 6)**	**(Ko8, *n* = 3)**
**Metabolic phenotypes**						
L-proline	+	+	−	+	+	−
D,L-a-Glycerol-phosphate	+	+	v	+	v	−
Alpha-Keto- Glutaric Acid	−	−	−	−	+	−
Glyoxylic Acid	−	−	−	v	−	−
Tricarballylic acid	+	+	−	+	+	−
Acetyl-b-D-Mannosamine	v	+	v	+	+	+
D-Melezitose	+	+	+	+	−	v
3-O-Methyl-Glucose	−	−	−	−	−	+
g-Amino-Butyric Acid	+	+	−	v	v	−
L-Tartaric Acid	v	v	v	+	+	−

*−, less than 20% of positive strains; +, more than 80% of positive strains; v, between 20 and 80% of positive strains.*

We also analyzed the MALDI-ToF MS peak patterns of the different members of the *K. oxytoca* complex. Based on the MALDI Biotyper Compass database version 4.1.80 (Bruker Daltonics, Bremen, Germany), the thirty strains were identified either as *K. oxytoca* (23 strains, all belonging to Ko1, Ko2, Ko4, and Ko6) or as *Raoultella ornithinolytica* (7 strains, all strains of Ko3 and Ko8). These misidentifications can be explained by the lack of reference spectra of most phylogroups in the reference database. Supplementary Figure S6 summarizes the peak positions found in each strain. A total of 31 biomarkers (2383–10152 *m/z*) associated with specific members of the *K. oxytoca* complex were identified (Supplementary Table S1 and Supplementary Figure S6). Consistent with genetic and biochemical findings, we also observed that Ko4 shared most of its spectral peaks with Ko1 and Ko6, presenting only one specific peak (which was variably present) at 3681 *m/z*, whereas Ko3 shared six peaks with only Ko8 and presented two unique peaks at 5178 and 6795 *m/z*. For the remaining phylogroups, specific peaks were observed for Ko2 and Ko8, whereas Ko1 and Ko6 could be identified by specific peak combinations. Based on the current dataset, the specificity and sensitivity of their distribution among phylogroups ranged between 60–100% and 80–100%, respectively (Supplementary Table S1). This finding paves the way to identify isolates of the *K. oxytoca* complex at the species (or phylogroup) level based on MALDI-ToF MS analysis, pending incorporation of reference spectra of the various taxa into reference spectra databases.

Based on the above genomic, phenotypic and proteomic characteristics, we propose Ko3 and Ko4 to be considered as two novel species, which we propose to name *K. spallanzanii* and *K. pasteurii*, respectively.

### Description of *Klebsiella spallanzanii* sp. nov.

*Klebsiella spallanzanii* (spal. lan.za ‘ni.i N. L. gen. n. referring to Lazzaro Spallanzani, Italian biologist, important contributor to the experimental study of bodily functions and of animal reproduction. He provided what is considered the first disproval of the theory of the spontaneous generation of microbes).

The description is based on 4 strains. Cells are Gram-negative, non-motile, non-spore-forming, straight, rod-shaped and capsulated. Colonies are smooth, circular, white, dome-shaped, and glistening. The general characteristics are as described for the genus *Klebsiella*. Indole-positive, ONPG-positive, lysine decarboxylase positive and ornithine decarboxylase negative. Differentiated from the other species of the *K. oxytoca* complex by the urease-positive (similar to Ko2) and Voges–Proskauer test negative (also negative for Ko8). Distinguished from the other members of *K. oxytoca* complex also by the characteristics listed in Table 3. Distinguishable from *K. huaxiensis* by the ability to use D-melezitose and the inability to ferment 3-O-methyl-glucose, and from the remaining *K. oxytoca* members by the inability to use L-proline. *K. spallanzanii* isolates were recovered from human urine and cow feces.

The type strain is strain SPARK_775_C1^T^ (=SB6411, CIP 111695T, DSM 109531T), isolated in 2017 from the urine of a patient in Pavia, Italy. The INSDC (GenBank/ENA/DDBJ) accession numbers of the *gyrA*, *rpoB* and *rrs* (coding for 16S rRNA) genes are MN076620, MN076626, and MN091365, respectively. The genome sequence accession number is ERS3550824. The DNA G + C content of the type strain is 53.3%.

### Description of *Klebsiella pasteurii* sp. nov.

*Klebsiella pasteurii* (pas. teu ‘ri.i N. L. gen. n. referring to Louis Pasteur, a French microbiologist, who made seminal contributions to microbiology and infectious diseases, vaccination and pasteurization. He contributed decisively to disprove the theory of the spontaneous generation of microbes).

The description is based on 14 strains. Cells are Gram-negative, non-motile, non-spore-forming, straight, rod-shaped and capsulated. Colonies are smooth, circular, white, dome-shaped, and glistening. The general characteristics are as described for the genus *Klebsiella*. Indole-positive, urease-negative, ONPG-positive, Voges–Proskauer test positive, lysine decarboxylase positive, and ornithine decarboxylase negative. They can be distinguished from the other members of *K. oxytoca* complex by the characteristics listed in Table 3. They are distinguishable from *K. grimontii* by the ability to ferment D-melezitose and inability to ferment alpha-keto-glutaric acid, and from the remaining *K. oxytoca* groups by the unique weak ability to ferment glyoxylic acid. *K. pasteurii* isolates were recovered from feces of cows, turtles and humans.

The type strain is strain SPARK_836_C1^T^ (=SB6412, CIP 111696T, and DSM 109530), isolated in 2017 from the feces of a patient in Pavia, Italy. The INSDC (GenBank/ENA/DDBJ) accession numbers of the *gyrA*, *rpoB*, and *rrs* (coding for 16S rRNA) genes are MN076619, MN076625 and MN091366, respectively. The genome sequence accession number is ERS3550825. The DNA G + C content of the type strain is 55.3%.

## Data Availability Statement

The nucleotide sequences generated in this study were deposited in European Nucleotide Archive (ENA) and are available through the INSDC databases under accession numbers MN091365 (SB6411T = SPARK775C1T), MN091366 (SB6412T = SPARK836C1T), MN104661 to MN104677 (16S rRNA), MN076606 to MN076643 (gyrA and rpoB), and MN030558 to MN030567 (blaOXY). Complete genomic sequences were submitted to European Nucleotide Archive under the BioProject number PRJEB15325.

## Ethics Statement

The approval of Ethical Committee of the San Matteo Hospital in Pavia was granted under number 20170001787 in date 25/05/2017, proceeding number is 2017000759. The internal code of the project is 0890170117. The ethical procedure includes written informed consent from all the patients participating in the study.

## Author Contributions

CM, MC, PM, CB, and DS isolated *Klebsiella* from diverse sources. CM, CR, VP, and MC performed the microbiological characterization of isolates. CM, HT, TK, and DS performed the genomic sequencing. CM, CR, HT, and TK analyzed the sequence data. CR and VP performed the MALDI-TOF analyses. VP and SB performed the phenotypic microarray analyses. CM, CR, and SB wrote the initial version of the manuscript. All authors revised the manuscript. EF, SB, JC, CB, and DS acquired funding for this study.

## Conflict of Interest

The authors declare that the research was conducted in the absence of any commercial or financial relationships that could be construed as a potential conflict of interest.

## Footnotes

1 http://ggdc.dsmz.de

2 https://github.com/tseemann/barrnap

3 https://bigsdb.pasteur.fr/klebsiella

## Supplementary Material

FIGURE S1 Phylogenetic relationships (neighbor-joining method, Jukes-Cantor correction) based on the aligned sequences of *gyrA* gene. The tree was rooted using *K. pneumoniae* DSM 30104^T^ (=ATCC 13883^T^). Taxonomic groups are indicated in front of the branches. Bootstrap proportions obtained after 1000 replicates are indicated at the nodes. Branch lengths represent the number of nucleotide substitutions per site (scale, 0.01 substitution per site). Strain labels are given as Strain Bank ID (e.g., SB73) followed by original strain name, followed by phylogroup. A “T” after the strain name indicates that the strain is the type strain of its taxon.

FIGURE S2 Phylogenetic relationships (neighbor-joining method, Jukes-Cantor correction) based on the aligned sequences of *rpoB* gene. The tree was rooted using *K. pneumoniae* DSM 30104^T^ (=ATCC 13883^T^). Taxonomic groups are indicated in front of the branches. Bootstrap proportions obtained after 1000 replicates are indicated at the nodes. Branch lengths represent the number of nucleotide substitutions per site (scale, 0.05 substitution per site). Strain labels are given as Strain Bank ID (e.g., SB73) followed by original strain name, followed by phylogroup. A “T” after the strain name indicates that the strain is the type strain of its taxon.

FIGURE S3 Phylogenetic tree based on chromosomal OXY beta-lactamase amino-acid sequences (maximum-likelihood method, Jones-Taylor-Thornton model). Branch lengths represent the number amino-acid substitutions per site (scale, 0.01 substitution per site). Taxonomic groups are indicated in front of the branches. Strain labels are given as Strain Bank ID (*e.g*., SB73) followed by original strain name, phylogroup, and the OXY beta-lactamase variant. A “T” after the strain name indicates type strains. Node labels indicate bootstrap values based on 1000 replicates.

FIGURE S4 Phylogenetic relationships (neighbor-joining method, Jukes-Cantor correction) based on the sequence of the *rrs* gene coding for 16S rRNA, and the respective multiple sequence alignment restricted to the variable positions. The tree was rooted using *K. pneumoniae* DSM 30104^T^ (=ATCC 13883^T^). Bootstrap proportions obtained after 1000 replicates are indicated at the nodes. Branch lengths represent the number of nucleotide substitutions per site (scale, 0.001 substitution per site). Strain labels are given as Strain Bank ID (e.g., SB73) followed by original strain name, followed by phylogroup. A “T” after the strain name indicates that the strain is the type strain of its taxon.

FIGURE S5 Metabolic phenotypes for the most discriminant carbon sources among members of the *K. oxytoca* complex. Black squares correspond to substrate utilization; white square to absence of utilization; and gray squares to intermediate values.

FIGURE S6 Peak positions (*m/z*) observed for each strain. Stars denote the peaks that are useful for discrimination among phylogroups, as detailed in Supplementary Table S1. ^1^Strains added for MALDI-ToF experiments.

TABLE S1 MALDI-ToF mass spectrometry peaks, which are useful biomarkers to discriminate phylogroups of the *Klebsiella oxytoca* species complex. CI, confidence interval. ^1^Position in the spectra using. ^2^Double-charged ion.

## Abbreviations

ANI: average nucleotide identity
HCCA: a-cyano -4-hydroxycinnamic acid
isDDH: *in silico* DNA-DNA hybridization
SCAI: simmons citrate agar with inositol
MALDI-ToF MS: matrix-assisted laser desorption/ionization time of flight mass spectrometry.
