# Supplementary material for: Description of Klebsiella spallanzanii sp. nov. and of Klebsiella pasteurii sp. nov
Source: Front Microbiol. 2019 Oct 25;10:2360. doi: 10.3389/fmicb.2019.02360 (PMC6824210; doi:10.3389/fmicb.2019.02360)
Supplement: FIGURE S1 — Phylogenetic relationships (neighbor-joining method, Jukes-Cantor correction) based on the aligned sequences of gyrA gene. The tree was rooted using K. pneumoniae DSM 30104T (=ATCC 13883T). Taxonomic groups are indicated in front of the branches. Bootstrap proportions obtained after 1000 replicates are indicated at the nodes. Branch lengths represent the number of nucleotide substitutions per site (scale, 0.01 substitution per site). Strain labels are given as Strain Bank ID (e.g., SB73) followed by original strain name, followed by phylogroup. A “T” after the strain name indicates that the strain is the type strain of its taxon. [file Data_Sheet_1.pdf]

Figure S1.

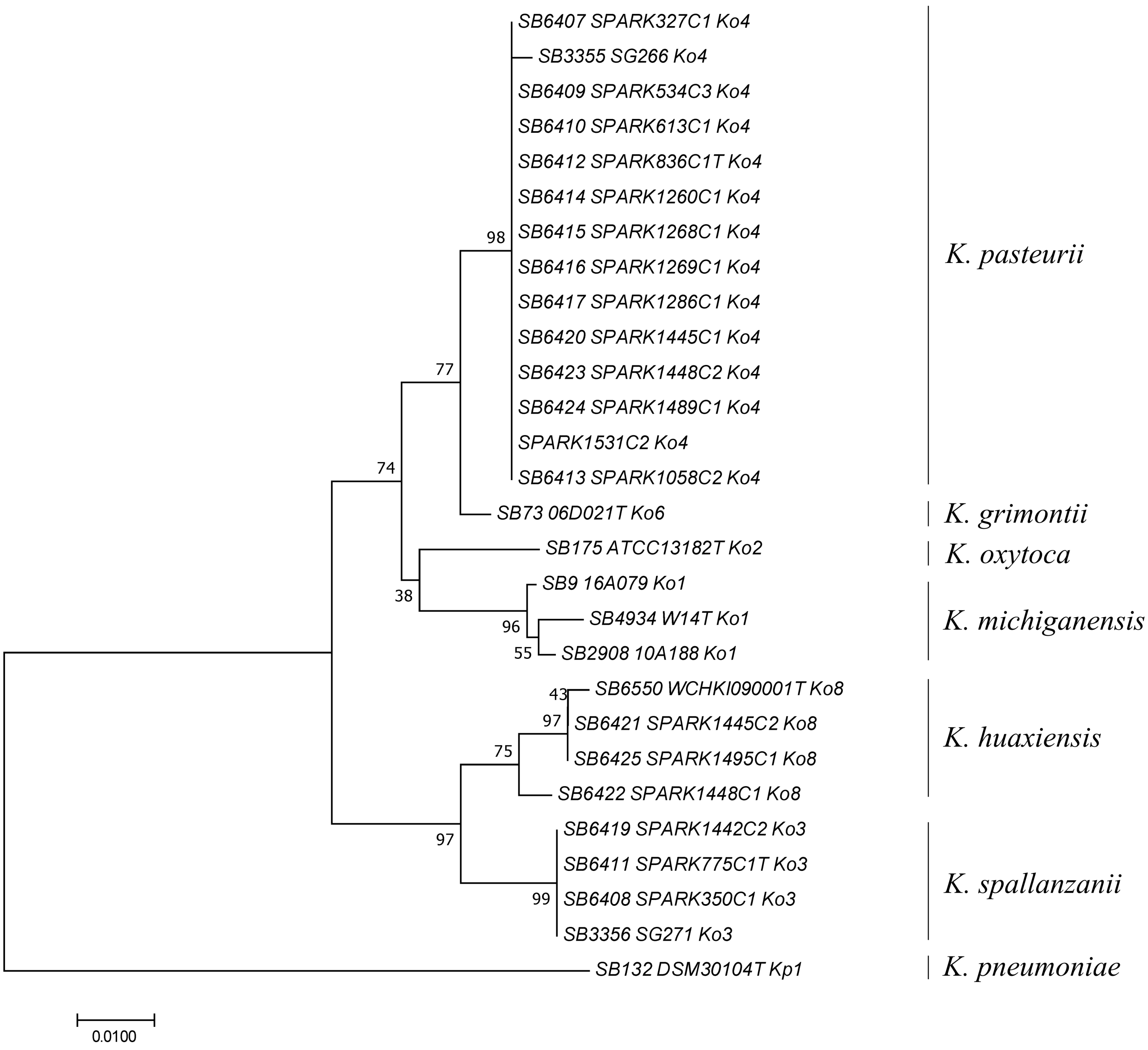
SB6407 SPARK327C1 Ko4
SB3355 SG266 Ko4
SB6409 SPARK534C3 Ko4
SB6410 SPARK613C1 Ko4
SB6412 SPARK836C1T Ko4
SB6414 SPARK1260C1 Ko4
SB6415 SPARK1268C1 Ko4
SB6416 SPARK1269C1 Ko4
SB6417 SPARK1286C1 Ko4
SB6420 SPARK1445C1 Ko4
SB6423 SPARK1448C2 Ko4
SB6424 SPARK1489C1 Ko4
SPARK1531C2 Ko4
SB6413 SPARK1058C2 Ko4
SB73 06D021T Ko6
SB175 ATCC13182T Ko2
SB9 16A079 Ko1
SB4934 W14T Ko1
SB2908 10A188 Ko1
SB6550 WCHKI090001T Ko8
SB6421 SPARK1445C2 Ko8
SB6425 SPARK1495C1 Ko8
SB6422 SPARK1448C1 Ko8
SB6419 SPARK1442C2 Ko3
SB6411 SPARK775C1T Ko3
SB6408 SPARK350C1 Ko3
SB3356 SG271 Ko3
SB132 DSM30104T Kp1
98
77
74
38
96
55
43
97
75
97
99
K. pasteurii
K. grimontii
K. oxytoca
K. michiganensis
K. huaxiensis
K. spallanzanii
K. pneumoniae
0.0100

Figure S2.

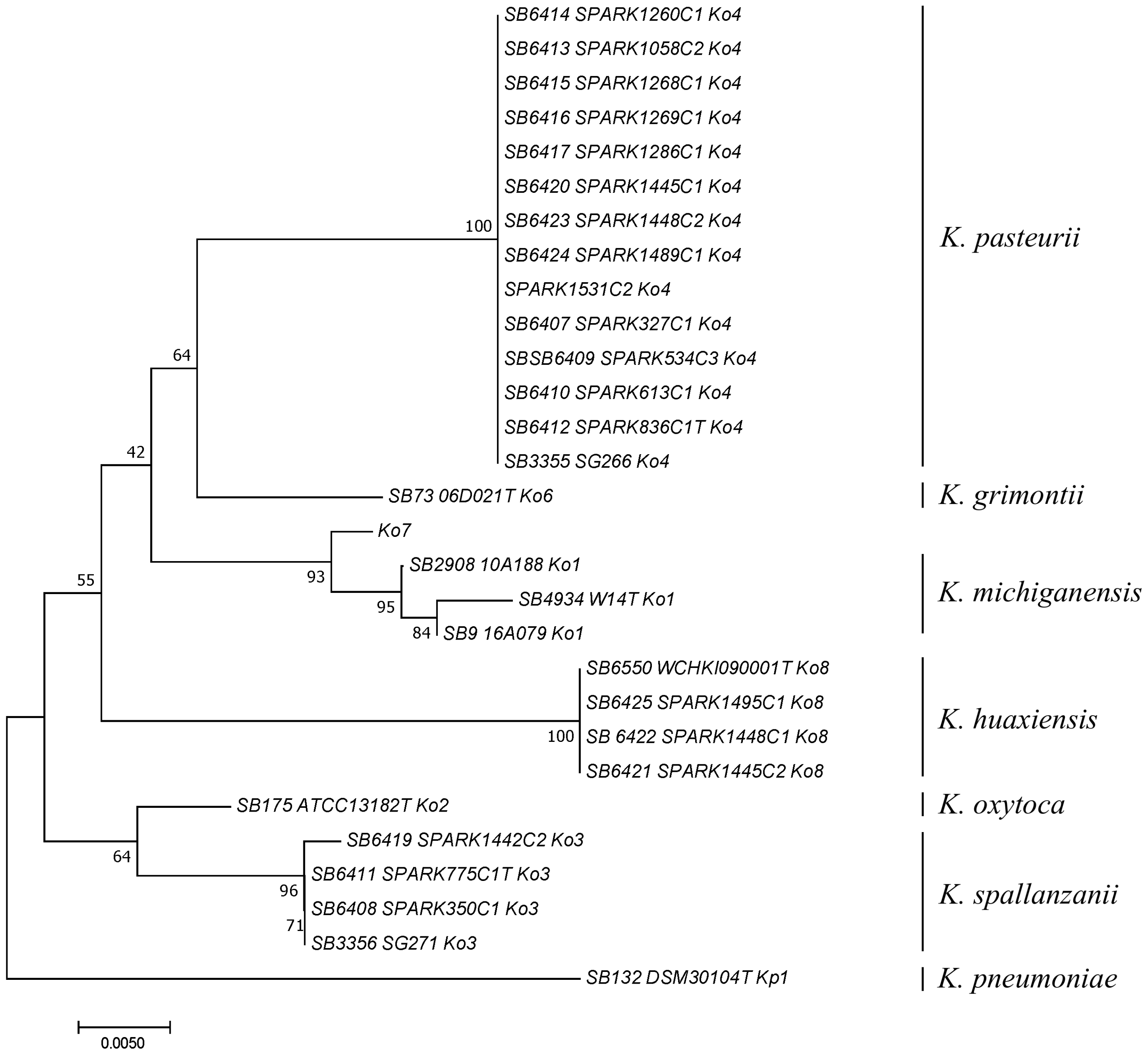
SB6414 SPARK1260C1 Ko4
SB6413 SPARK1058C2 Ko4
SB6415 SPARK1268C1 Ko4
SB6416 SPARK1269C1 Ko4
SB6417 SPARK1286C1 Ko4
SB6420 SPARK1445C1 Ko4
SB6423 SPARK1448C2 Ko4
SB6424 SPARK1489C1 Ko4
SPARK1531C2 Ko4
SB6407 SPARK327C1 Ko4
SBSB6409 SPARK534C3 Ko4
SB6410 SPARK613C1 Ko4
SB6412 SPARK836C1T Ko4
SB3355 SG266 Ko4
K. pasteurii
100
64
42
SB73 06D021T Ko6
K. grimontii
Ko7
SB2908 10A188 Ko1
SB4934 W14T Ko1
SB9 16A079 Ko1
K. michiganensis
93
95
84
55
SB6550 WCHKI090001T Ko8
SB6425 SPARK1495C1 Ko8
SB 6422 SPARK1448C1 Ko8
SB6421 SPARK1445C2 Ko8
K. huaxiensis
100
SB175 ATCC13182T Ko2
K. oxytoca
SB6419 SPARK1442C2 Ko3
SB6411 SPARK775C1T Ko3
SB6408 SPARK350C1 Ko3
SB3356 SG271 Ko3
K. spallanzanii
64
96
71
SB132 DSM30104T Kp1
K. pneumoniae
0.0050

Figure S3.

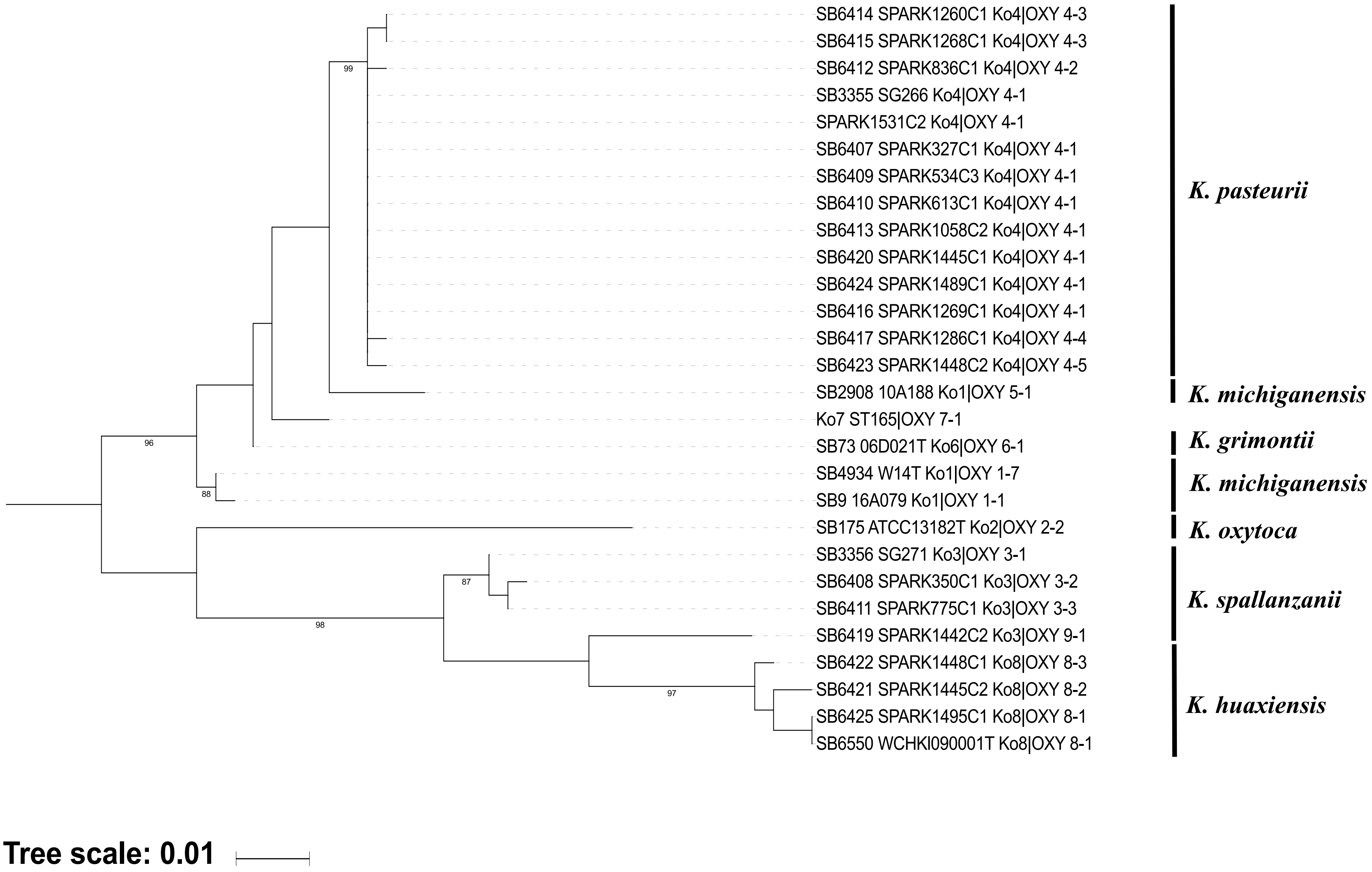
SB6414 SPARK1260C1 Ko4|OXY 4-3
SB6415 SPARK1268C1 Ko4|OXY 4-3
SB6412 SPARK836C1 Ko4|OXY 4-2
SB3355 SG266 Ko4|OXY 4-1
SPARK1531C2 Ko4|OXY 4-1
SB6407 SPARK327C1 Ko4|OXY 4-1
SB6409 SPARK534C3 Ko4|OXY 4-1
SB6410 SPARK613C1 Ko4|OXY 4-1
SB6413 SPARK1058C2 Ko4|OXY 4-1
SB6420 SPARK1445C1 Ko4|OXY 4-1
SB6424 SPARK1489C1 Ko4|OXY 4-1
SB6416 SPARK1269C1 Ko4|OXY 4-1
SB6417 SPARK1286C1 Ko4|OXY 4-4
SB6423 SPARK1448C2 Ko4|OXY 4-5
SB2908 10A188 Ko1|OXY 5-1
Ko7 ST165|OXY 7-1
SB73 06D021T Ko6|OXY 6-1
SB4934 W14T Ko1|OXY 1-7
SB9 16A079 Ko1|OXY 1-1
SB175 ATCC13182T Ko2|OXY 2-2
SB3356 SG271 Ko3|OXY 3-1
SB6408 SPARK350C1 Ko3|OXY 3-2
SB6411 SPARK775C1 Ko3|OXY 3-3
SB6419 SPARK1442C2 Ko3|OXY 9-1
SB6422 SPARK1448C1 Ko8|OXY 8-3
SB6421 SPARK1445C2 Ko8|OXY 8-2
SB6425 SPARK1495C1 Ko8|OXY 8-1
SB6550 WCHKI090001T Ko8|OXY 8-1
99
96
88
87
98
97
K. pasteurii
K. michiganensis
K. grimontii
K. michiganensis
K. oxytoca
K. spallanzanii
K. huaxiensis
Tree scale: 0.01

Figure S4.

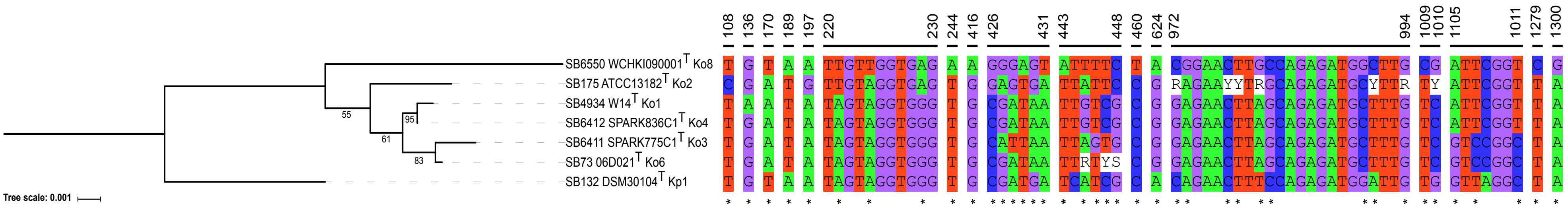
SB6550 WCHKI090001T Ko8
SB175 ATCC13182T Ko2
SB4934 W14T Ko1
SB6412 SPARK836C1T Ko4
SB6411 SPARK775C1T Ko3
SB73 06D021T Ko6
SB132 DSM30104T Kp1
Tree scale: 0.001
55
95
61
83

Figure S5.

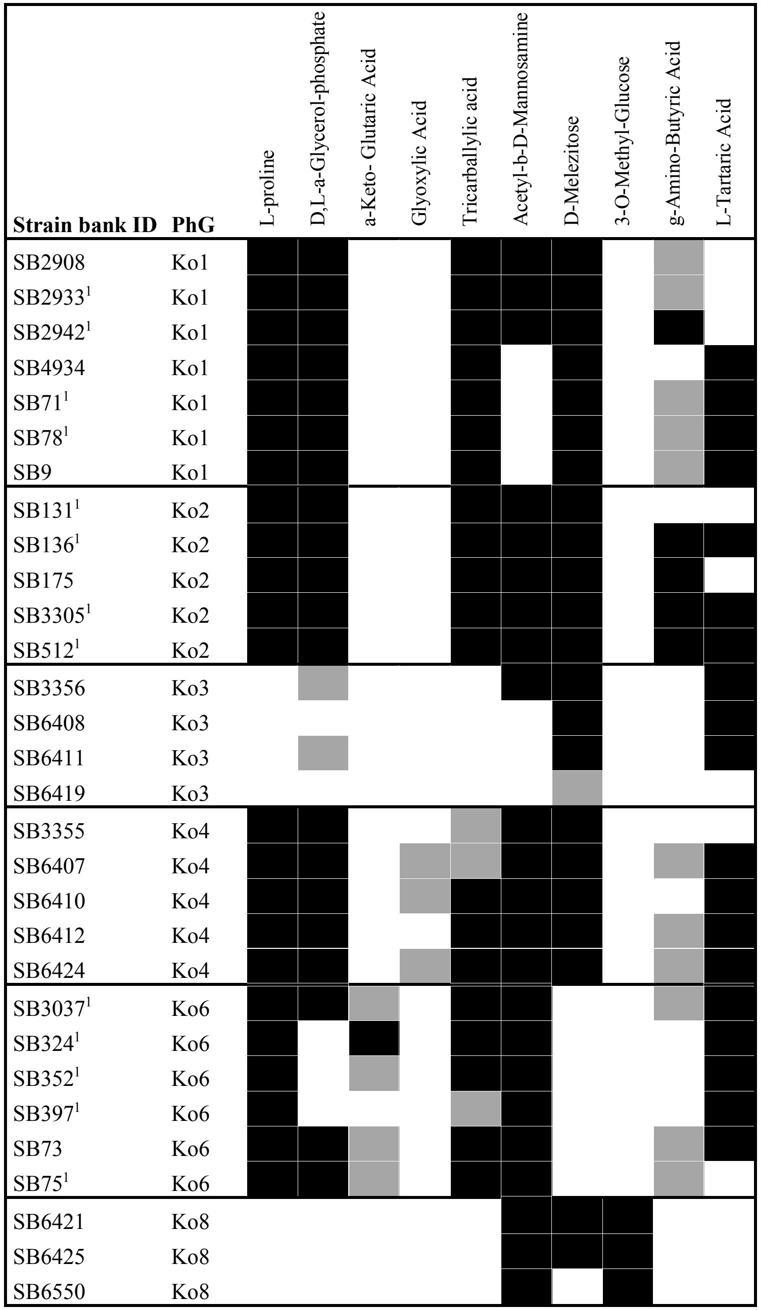

PhG, Phylogroup; [1]Strains added to the study for phenotype microarray experiments (Biolog)

Figure S6.

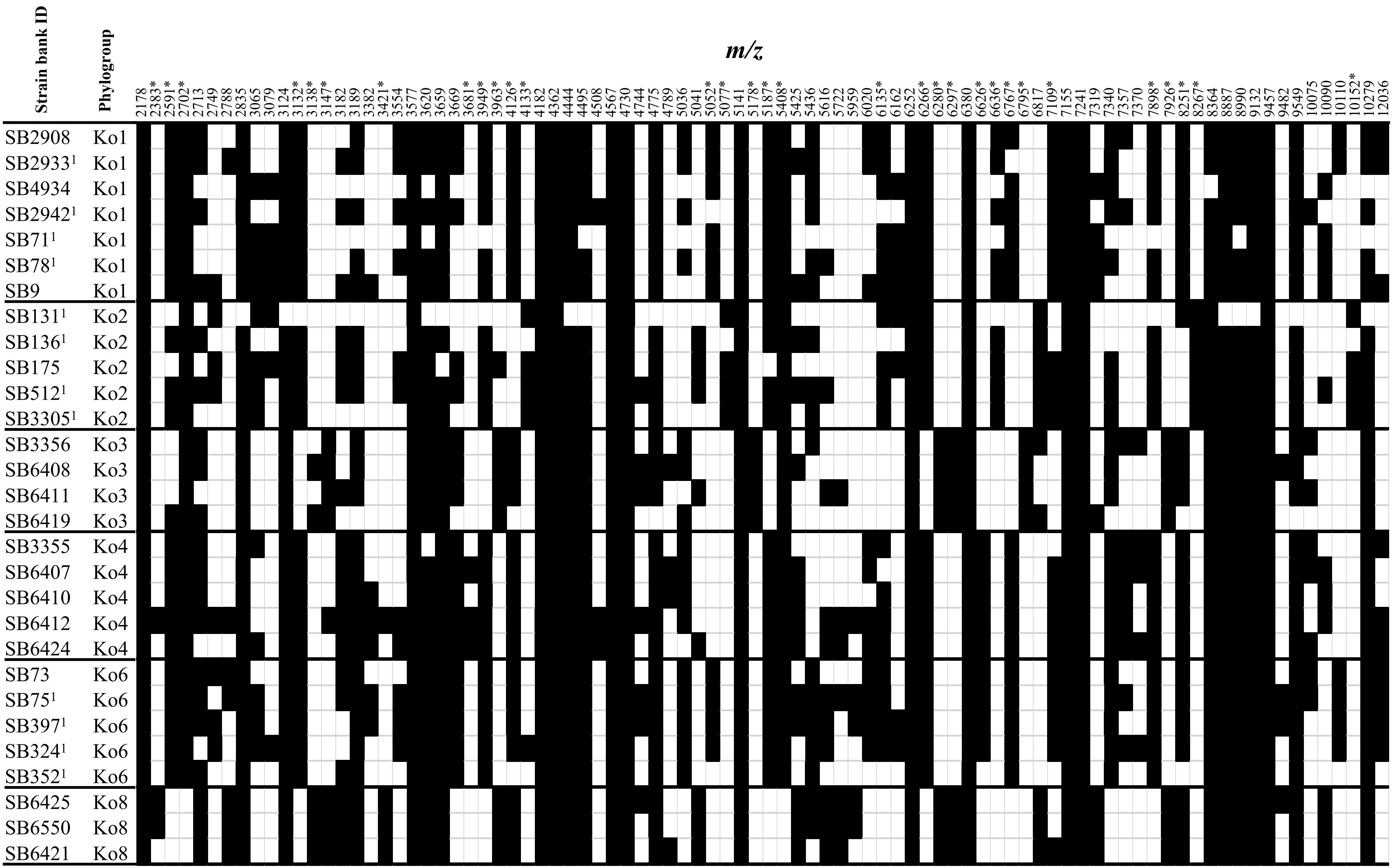
Strain bank ID
Phylogroup
m/z
2178 2383* 2591* 2702* 2713 2749 2788 2835 3065 3079 3124 3132* 3138* 3147* 3182 3189 3382 3421* 3554 3577 3620 3659 3669 3681* 3949* 3963* 4126* 4133* 4182 4362 4444 4495 4508 4567 4730 4744 4775 4789 5036 5041 5052* 5077* 5141 5178* 5187* 5408* 5425 5436 5616 5722 5959 6020 6135* 6162 6252 6266* 6280* 6297* 6380 6626* 6636* 6767* 6795* 6817 7109* 7155 7241 7319 7340 7357 7370 7898* 7926* 8251* 8267* 8364 8887 8990 9132 9457 9482 9549 10075 10090 10110 10152* 10279 12036
SB2908 Ko1
SB2933[1] Ko1
SB4934 Ko1
SB2942[1] Ko1
SB71[1] Ko1
SB78[1] Ko1
SB9 Ko1
SB131[1] Ko2
SB136[1] Ko2
SB175 Ko2
SB512[1] Ko2
SB3305[1] Ko2
SB3356 Ko3
SB6408 Ko3
SB6411 Ko3
SB6419 Ko3
SB3355 Ko4
SB6407 Ko4
SB6410 Ko4
SB6412 Ko4
SB6424 Ko4
SB73 Ko6
SB75[1] Ko6
SB397[1] Ko6
SB324[1] Ko6
SB352[1] Ko6
SB6425 Ko8
SB6550 Ko8
SB6421 Ko8

Table S1. MALDI-ToF mass spectrometry peaks, which are useful biomarkers to discriminate phylogroups of the *Klebsiella oxytoca* species complex.

**Phylogroup(s) in which the peak was observed**	**Peak Position** (*m/z*)[1]	**Sensitivity** [95% CI]	**Specificity** [95% CI]
**Ko1, Ko4, Ko6, Ko3, Ko2**	2702[2]	100% [86.77% - 100.00%]	100% [29.24% - 100.00%]
	5408	100% [86.77% - 100.00%]	100% [29.24% - 100.00%]
**Ko1, Ko4, Ko6, Ko3, Ko8**	4126[2]	91.67% [73.00% - 98.97%]	100% [47.82% - 100.00%]
	8251	91.67% [73.00% - 98.97%]	80% [28.36% - 99.49%]
**Ko1, Ko4, Ko6, Ko2**	2591[2]	90.91% [70.84% - 98.88%]	85.71% [42.13% - 99.64%]
	5187	95.45% [77.16% - 99.88%]	100% [59.04% - 100%]
	3132[2]	95.45% [77.16% - 99.88%]	100% [59.04% - 100%]
	6266	100% [84.56% - 100%]	100% [59.04% - 100%]
	3949[2]	90.91% [70.84% - 98.88%]	100% [59.04% - 100%]
	7898	90.91% [70.84% - 98.88%]	100% [59.04% - 100%]
	6135	81.82% [59.72% - 94.81%]	100% [59.04% - 100%]
	7109	81.82% [59.72% - 94.81%]	85.71% [42.13% - 99.64%]
	6767	94.12% [71.31% - 99.85%]	100% [73.54% - 100%]
**Ko1, Ko6**	5052	83.33% [51.59% - 97.91%]	100% [80.49% - 100.00%]
**Ko6, Ko4**	6626	90% [55.50% - 99.75%]	100% [82.35% - 100.00%]
**Ko4**	3681	60% [14.66% - 94.73%]	100% [85.75% - 100%]
**Ko1, Ko2**	6636	85.71% [42.13% - 99.64%]	100% [78.20% - 100%]
**Ko2**	4133[2]	80% [28.36% - 99.49%]	95.83% [78.88% - 99.88%]
	8267	100% [47.82% - 100.00%]	100% [85.75% - 100.00%]
	5077[2]	80% [28.36% - 99.49%]	100% [85.75% - 100.00%]
	10152	80% [28.36% - 99.49%]	100% [85.75% - 100.00%]
**Ko3, Ko8**	3138[2]	71.43% [29.04% - 96.33%]	100% [84.56% - 100%]
	6280	100% [59.04% - 100.00%]	100% [84.56% - 100%]
	3147[2]	100% [59.04% - 100.00%]	95.45% [77.16% - 99.88%]
	6297	100% [59.04% - 100.00%]	100% [84.56% - 100%]
	3963[2]	100% [59.04% - 100.00%]	96.15% [80.36% - 99.90%]
	7926	100% [59.04% - 100.00%]	100% [84.56% - 100%]
**Ko3**	5178	100% [39.76% - 100.00%]	100% [86.28% - 100%]
	6795	100% [39.76% - 100.00%]	100% [86.28% - 100%]
**Ko8**	2383	66.67% [9.43% - 99.16%]	96.15% [80.36% - 99.90%]
	3421	100% [29.24% - 100.00%]	96.15% [80.36% - 99.90%]

CI, confidence interval
[1] Position in the spectra using a tolerance of ±0.03%.
[2] Double-charged ion.
